# Diseases of the Circulation

**Published:** 1905-08-19

**Authors:** 


### DISEASES OF THE CIRCULATION.

### APOPLEXY.

**The Causes of Apoplexy.**—A great deal of work has recently been done on this subject, and the numerous discussions upon the causes of high tension have led to some useful results. Clifford Allbutt has in various papers taught us the necessity of recognising early stages of persistent high pressure, of measuring it by instruments more exact than the finger, of removing known causes, and of preventing a further rise when possible. He has shown, too, what is most important, that high pressures do often occur without granular kidneys, that arterio-sclerosis is not a cause but a result of such pressures, and that early treatment will often check the rise until the age when the tendency ceases.[1] It is true that many forms of "apoplexy" and hemiplegia are due not to cerebral hæmorrhage but to thrombosis and embolism. Unfortunately, little progress has of late been made in diagnosing and combating the conditions which lead to these disorders. We must here, therefore, confine ourselves to the papers on high tension and hæmorrhage. One of the most instructive is that by H. W. Cook of Johns Hopkins.[2] He lays down that "apoplexies" most frequently occur in vigorous men with nearly normal vessels, but with hypertension, while far older persons whose arteries feel like beaded pipe stems rarely suffer. From the results of this high tension two-thirds of the males in some families regularly die before the age of 60, the most dangerous period perhaps being that about 50 years. He agrees with Allbutt that chronic high tension is rarely produced by sclerosis, nor does it lead to sclerosis, but rather to tortuous and thickened arteries. The causes are some forms of lung disease such as emphysema, cardiac hypertrophy from excessive labour or exertion as in worn-out athletes, and states where compensation has followed after valvular incompetency. It is produced, too, by the excessive use of tobacco when inhaled, but not directly by alcohol, though alcoholism may cause intestinal toxins which lead to it. This is confirmed by Cabot, who finds that alcohol of itself produces no marked or permanent rise of pressure, and thinks that it does not even produce arterio-sclerosis. Other causes of high tension are the toxins present in nephritis, eclampsia, diabetes, and plumbism. It also follows attacks of gout, syphilis, typhoid, and other febrile disorders, and finally it occurs in many instances of quite unknown causation. In some families there seems to be an hereditary tendency to the disease, though the sufferers may be of most careful habits, and on the other hand, many instances are found in very strong plethoric individuals with huge appetities, and again in chronic dyspeptics of sedentary habits. Very notable too are the numbers affected of professional men who undergo great mental strain and anxiety. J. Barr [3] thinks that an underlying cause is sometimes a deficient action of the thyroid and over-action of the suprarenal bodies, or an excess of purin bodies in the organism, while Stengel agrees with Allbutt in referring most cases to overwork, overeating and drinking, or to the absorption of toxins in certain diseases or by certain metallic poisons such as lead. An interesting question has been raised as to the effect of changes in atmospheric pressure on persons suffering from such excessive tension: Are they more liable to cerebral hæmorrhage when the barometric pressure is high, and how does residence at a high altitude act upon them. J. W. Russell [4] has examined the weather records on the days when attacks occurred in a large number of patients at Liverpool and compared the pressures and temperatures. The result was that no connection could be traced between the incidence of attacks and the atmospheric temperature, the barometric or wind pressure. We may conclude, then, that the most varied causes produce the condition and that while some are well known there are others whose existence can only be surmised. Some of them clearly depend on vasomotor alterations and some, probably, on changes in viscosity.

**The Prevention of Cerebral Hæmorrhage.**—In considering the subject the first question must be, can we get rid of any poison such as that due to Bright's disease which sets up the high tension? and, secondly, can we reduce excessive tension and maintain the vessels in a healthy condition? Now, in the large class of cases where Bright's disease is not present, Allbutt's warning is a sound one—namely, to test the blood-pressure accurately and at an early stage, and, if it is excessive, to restrict the patient's food, drink, and work, to give occasional courses of arsenic and the nitrites, and from time to time mercurials and salines. If we could permanently lower a dangerously high tension by the use of the nitrites, our task would be easier, but their action is so brief that doubts occur as to their value. Still, the frequent but temporary relief of nitroglycerine seems to result in freeing the patient from his symptoms for months or years. H. P. Loomis [5] disputed the value of the drug in causing even a temporary lowering in chronic hypertension, but G. B. Wallace, in reply, gave an instance where it produced a fall of 150 mm., though he confessed that in the paroxysms of angina it sometimes gives little relief without the simultaneous use of morphia or chloroform. The effect of sodium nitrite and erythrol is undoubtedly more prolonged. Oliver has recently recommended ammonium hippurate in two-grain doses, as having a prolonged effect. Barr praises thyroid extract and the iodine compounds as vaso-dilators. The reason for the use of the iodides is not clear, and we recall the old disputes as to its action upon aneurysms. Romberg, however, thinks it diminishes viscosity. In practice a course of iodide occasionally seems to have some value, but we must not rest content with a general sense of improvement, for in the sphygmometer, at least in its later forms, we have now an exact and accurate measure of the reduction of tension, which ought to largely replace the finger in estimating a matter of such importance. A false estimate may result in our patient being killed or crippled for life by a sudden hæmorrhage, and when the pulse is rapid and "small"—that is, when the difference between the diastolic and systolic pressures is little, the finger is very apt to give an impression of quite a low pressure. C. J. Martin [6] showed experimentally that, with a good instrument, we can determine the true internal pressure within four per cent., and that, even where the vessel walls are greatly sclerosed, the readings do not differ more than 7 mm. from the true amount. The tension then should be carefully estimated from time to time, the diet, the fluid taken and the work undergone must be regulated. Freely acting aperients, such as a blue pill and salines, are of the highest value, and a course of one of the vaso-dilators should be given whenever the tension begins to rise, though we have to bear in mind that we do not require to reduce the tension below a limit which is consistent with safety. Success is confessedly uncertain. In some cases the machine can only be run at high pressure, in others it is difficult to eliminate the poison which causes the hypertension, and in others we are absolutely ignorant what the cause of it is.

[1] Bristol Med. Chir. Jour., March. [2] Jour. Amer. Med. Assoc., p. 203, 1905. [3] Brit. Med. Jour., Jan. 14. [4] Lancet, April 22. [5] Med. Rec., Feb. 28 [6] Brit. Med. Jour., Jan. 14.

